# Epidemiological and clinical differences of coronavirus disease 2019 patients with distinct viral exposure history

**DOI:** 10.1080/21505594.2020.1802870

**Published:** 2020-08-12

**Authors:** Yinjie Gao, Xuemei Ma, Jingfeng Bi, Jindong Chu, Bo Liu, Chunsheng Chi, Jianguo Yan, Xiaoli Yu, Fangming Liu, Xiaohong Deng, Hongbing Zhang, Bo Jin

**Affiliations:** a Liver Transplantation and Research Center, Department of Liver Cirrhosis Diagnosis and Treatment Centre, Research Centre for Clinical and Translational Medicine, the Fifth Medical Center, Chinese PLA General Hospital and National Clinical Research Center for Infectious Diseases, Beijing, China; b State Key Laboratory of Medical Molecular Biology, Department of Physiology, Institute of Basic Medical Sciences, Chinese Academy of Medical Sciences and Peking Union Medical College, Beijing, China; c Beijing Menlobiotech, Beijing, China

**Keywords:** Coronavirus, SARS-CoV-2, Covid-19, incubation period, pandemic, virulence

## Abstract

Since severe acute respiratory syndrome coronavirus 2 (SARS-CoV-2) spread from the early epicenter, Wuhan, to the rest of China, the virulence of SARS-CoV-2 might have evolved at different phases of the pandemic. We therefore compared the unique features among 62 coronavirus disease 2019 (COVID-19) inpatients who contracted SARS-CoV-2 in Wuhan (15 cases), exposed to the patients from Wuhan (26 cases), or acquired the disease without exposure to Wuhan patients (21 cases). Median incubation periods are 4.5 days (3–5) for Wuhan patients, 8 days (3–11) for those infected by Wuhan patients, and 12 days (7–13) for those without aforementioned experience. The disease onset dates are earliest for Wuhan patients and latest for those without exposure to Wuhan patients. Blood lymphocytes were lowest in Wuhan patients, lower in those affected by Wuhan patients, and modest reduced in remaining ones. Disease severity is worst for Wuhan patients, and modest for those without contact with Wuhan patients. Wuhan patients had longest (27 days, 18–28), those transmitted by Wuhan patients had intermediate (16 days, 8–23), and the rest of the patients had shortest (13 days, 8.5–22.5) hospital stay. Early viral exposure, older age, lymphocytopenia, and underlying conditions are risk factors which warrant aggressive intervention. Even though the virulence of SARS-CoV-2 appears decline over the course of serial transmissions, viral testing, contact tracing, social distancing, and face masking should be imposed on general public to contain viral dissemination from both symptomatic and asymptomatic patients with this highly contagious disease.

## Introduction

An outbreak of respiratory illness caused by a novel RNA virus of *Coronaviridae* family was reported in Wuhan, Hubei, China [1–3]. Considering it a sister to severe acute respiratory syndrome coronaviruses (SARS-CoVs), International Committee on Taxonomy of Viruses assigned this novel virus as severe acute respiratory syndrome coronavirus 2 (SARS-CoV-2) [4]. The World Health Organization (WHO) designated this disease as coronavirus disease 2019 (COVID-19) [5]. Because SARS-CoV-2 has plagued over 210 countries/territories/areas, WHO considered the public health threat posed by COVID-19 very high globally. As of 16 July 2020, WHO reported 13,378,853 confirmed cases and 580,045 deaths worldwide [6]. Due to exponential increase in cases and deaths, the US has become the country with the most confirmed cases and deaths of COVID-19. This widespread person-to-person transmission of SARS-CoV-2 has thus become a serious pandemic to threat public health globally. Navigating our responses in this uncharted arena and mitigating acquisition of viral infection and disease progression are global challenges.

China had 84,373 confirmed cases and 4,643 deaths with majority of them in Wuhan (50,333; 3,869) by 30 April 2020 [7]. To contain the rapid spread of SARS-CoV-2, Wuhan sealed its borders from the rest of China on 23 January 2020. The potential differences of epidemiological feature, clinical manifestation, response to medical intervention, and patient prognosis are unknown between patients with early infection inside Wuhan and subsequent transmissions beyond Wuhan. No direct comparison of earlier infected patients with later transmitted ones in the same medical setting(s) was reported. To elucidate the shared features and unique characteristics between these who infected with the virus in Wuhan before lockdown to those who contracted the virus later outside Wuhan, we retrospectively analyzed 62 COVID-19 inpatients treated from 20 January 2020 to 17 March 2020 at the Fifth Medical Center, Chinese PLA General Hospital, a designated medical center for care of COVID-19 patients in Beijing of China.

## Methods

### Study design and patients

This study was approved by the Medical Ethics Review Committee of the Fifth Medical Center, Chinese PLA General Hospital (2020018D). For this retrospective cohort study, we analyzed 62 Chinese patients with COVID-19 from 20 January to 17 March  2020 admitted at the Fifth Medical Center, Chinese PLA General Hospital. Pharyngeal swab specimens were obtained from all patients at admission and sent to Beijing Center for Disease Control for laboratory diagnosis. COVID-19 was diagnosed according to the WHO interim guidance and confirmed by at least two positive real-time reverse-transcriptase polymerase-chain-reaction (RT-PCR) tests for viral nucleic acids [8–10].

We designated 24 January 2020 as illness onset cutoff date after lockdown of Wuhan implemented on 23 January 2020. We set patients’ exposure history into three periods: (1) patients contracted the virus in Wuhan (Wuhan); (2) those exposed to Wuhan patients outside of Wuhan (Wuhan-related); and those acquired the disease outside of Wuhan without exposure to Wuhan patients (non-Wuhan). The clinical classifications are as follows [1]: Mild, The clinical symptoms are mild and no pneumonia manifestation can be found in imaging [2]. Ordinary, Patients have symptoms and pneumonia manifestation can be seen in imaging [3]. Severe, Meet any of the following: (a) Respiratory distress, RR≥30 breaths/min; (b) pulse oxygen saturation (SpO_2_)≤93% on room air at rest state; (c) arterial partial pressure of oxygen (PaO_2_)/oxygen concentration (FiO_2_)≤300 mmHg. Patients with >50% lesions progression within 24–48 hours in pulmonary imaging should be treated as severe cases [4]. Critical, Meeting any of the following: (a) respiratory failure occurs and mechanical ventilation is required; (b) shock occurs; (c) complicated with other organ failure that requires monitoring and treatment in intensive care unit. We cataloged mild and ordinary patients into nonsevere group and severe and critical patients into severe group on admission. Discharge criteria are as follows [1]: with normal body temperature for more than 3 days [2]; with significantly recovered respiratory symptoms [3]; lung imaging shows obvious absorption and recovery of acute exudative lesion [4]; virus clearance was evidenced by negative results of the nucleic acid tests of SARS-CoV-2 for consecutive two times (sampling interval at least 1 day) [11].

### Data collection

Epidemiology, comorbidity, symptoms, vital signs, laboratory tests (complete blood count, blood chemical analysis, C-reactive protein [CRP], Procalcitonin [PCT], Interlukin-6 [IL-6], and Pro-brain natriuretic peptide [BNP]), chest computed tomographic (CT) scans, treatment measures such as antiviral, antibiotics, corticosteroid, respiratory support, and prognosis were all obtained from hospital electronic medical records. The incubation period was defined as the time from earliest possible exposure to the infected patients to the onset of illness. All the data were analyzed by a group of highly trained physicians and scientists.

### Statistical analysis

Categorical variables were expressed as counts and percentages and analyzed using chi-square tests (sample size≥40 and expected frequency≥5) or Fisher’s test (sample size<40 or Expected frequency <5), corrected α’ = α/[k(k-1)/2] if comparing pairs within a group. Normality tests were conducted using Shapiro-Wilk test. Continuous variables were reported as the mean ± SD for normality and homogeneity of variances or median and interquartile range [IQR] for non-normality or non-homogeneity of variances. Continuous variables were compared using ANOVA for 3-group (SNK-q tests between-group comparisons) and *t* test for two-group comparison for normality and homogeneity of variances. With non-normality or non-homogeneity of variances, Kruskal–Wallis was used for three-group (Wilcoxon rank sum test if comparing pairs within a group, corrected α’ = α/[k(k-1)/2]) and Wilcoxon rank sum test for two-group comparison. Multiple linear regression examined how multiple independent variables were related to one dependent variable. Statistical analyses were performed with SAS software, version 9.4 (Cary, NC, USA), the statistical significance is defined as a two-sided α = 0.05.

## Results

### Demographic and epidemiological characteristics of patients infected at different phases of COVID-19 outbreak

**Figure 1.**
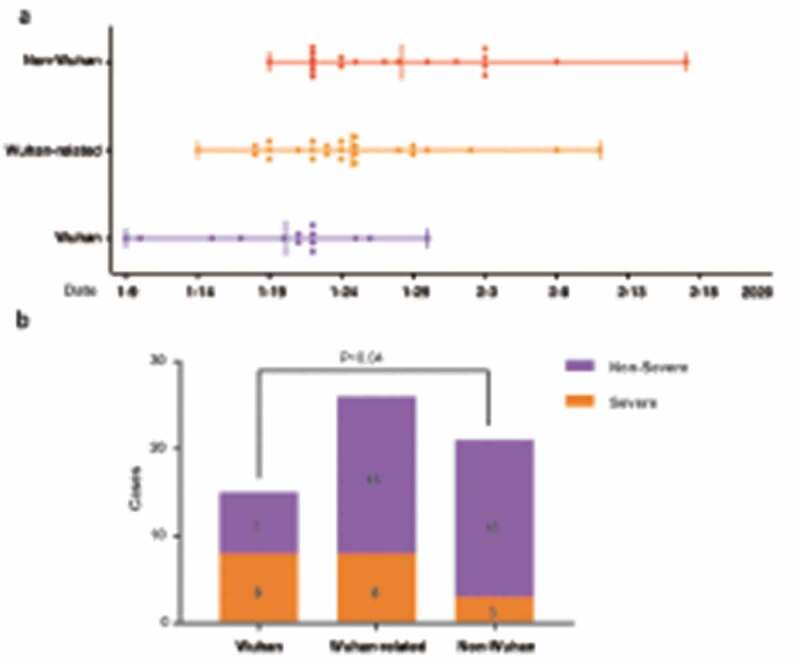
Disease onset date and severity of COVID-19 patients. (a). Distribution of disease onset dates. (b). Disease severity. Wuhan: patients were infected in Wuhan; Wuhan-related: patients contracted the virus from Wuhan patients outside of Wuhan; non-Wuhan: patients acquired the disease outside of Wuhan without exposure to Wuhan patients.

From January 20 to 17 March 2020, 62 confirmed COVID-19 patients were hospitalized. The median age of the patients was 48.3 years, 34 (55%) patients were men. Patients infected by SARS-Cov-2 viruses in Wuhan before lockdown on 23 January 2020 were more likely acquired the viruses during early stage of the outbreak, while those infected outside Wuhan ought to be sicken by subsequent viral transmission. Of 62 patients, 15 (24%) patients were infected in Wuhan, 26 (42%) contracted the virus from Wuhan patients, and 21 (32%) acquired the disease without exposure to Wuhan patients. We therefore designated them as Wuhan, Wuhan-related, and non-Wuhan patients, respectively. Age, gender and preexisting comorbidities were similar among these three groups (Table 1). Disease onset was emerged in 12 (80%) Wuhan, 12 (46%) Wuhan-related, and 7 (33%) non-Wuhan patients before Wuhan lockdown. The disease onset dates of this cohort distributed roughly in early, middle, and late periods for Wuhan, Wuhan-related, and non-Wuhan patients (Table 1, Figure 1(a)). The median incubation periods of COVID-19 are estimated as 4.5 days [3–5] for Wuhan, 8 days [3–11] for Wuhan-related, and 12 days [7–13] for non-Wuhan patients. They were admitted to the hospital 5 days [3–8], 4 days [3–9], and 5.5 days (2.5–8.5), respectively, after disease onset (Table 1).

**Table 1.** Demographic and clinical features of COVID-19 patients with various viral exposure histories.

	Wuhan [15]	Wuhan-related [26]	Non-Wuhan [21]	P value			
				ANONA	Wuhan vs non-Wuhan	Wuhan vs Wuhan-related	Wuhan-related vs non-Wuhan
^1^Age-yr	50 (37–55)	46 (35–59)	46 (37–66)	0.8857	0.9105	0.7452	0.6300
^2^Sex-no. (%)	0.2791				0.5160	0.1912	0.3896
Female	9 (60.0)	9 (34.6)	10 (47.6)				
Male	6 (40)	17 (65.4)	11 (52.4)				
^2^Onset-no. (%)				*0.0255*	*0.0082*	0.4463	0.0341
Before 01/24/2020	12 (80.0)	12 (46.1)	7 (33.3)				
After 01/24/2020	3 (20.0)	14 (53.9)	13 (69.7)				
^1^Incubation period, days	4.5 (3–5)	8 (3–11)	12 (7–13)	*0.0014*	*0.0003*	0.0611	0.0436
^1^Onset to admission, days	5 (3–8)	4 (3–9)	5.5 (2.5–8.5)	0.717	0.5024	0.8215	0.4238
^2^Disease severity-no. (%)	*0.0432*	*0.0122*	0.1537	0.3000			
Nonsevere	7 (46.7)	18 (69.2)	18 (85.7)				
Severe	8 (53.3)	8 (30.8)	3 (14.3)				
^2^Comorbidities-no. (%)							
Respiratory disease	1 (6.7)	3 (11.5)	0 (0)	0.3467	0.4167	1.000	0.2424
Hypertension	4 (26.7)	5 (19.2)	7 (33.3)	0.5332	0.7288	0.7010	0.3258
Diabetes	2 (13.3)	5 (19.2)	2 (9.5)	0.739	1.000	1.000	0.4364
Cardiovascular disease	0 (0)	1 (3.8)	3 (14.3)	0.2768	0.2500	1.000	0.3112
Chronic kidney disease	0 (0)	1 (3.8)	1 (4.8)	1.000	1.000	1.000	1.000
^2^Symptoms-no. (%)							
Fever	14 (93.3)	24 (92.3)	17 (81.0)	0.4597	0.3761	1.000	0.3856
Cough	11 (73.3)	16 (61.5))	11 (52.4)	0.4448	0.2036	0.4430	0.5279
Expectoration	8 (53.3)	6 (23.1)	2 (9.5)	*0.0141*	*0.0071*	0.0491	0.2690
Dyspnea	7 (46.7)	4 (15.4)	4 (19.0)	0.0772	0.1410	0.0639	1.0000
^2^Chest CT-no. (%)							
Ground-glass opacity	15 (100.0)	17 (65.4)	15 (71.4)	*0.0255*	0.0304	*0.0154*	0.6585
Bilateral patchy shadowing	11 (73.3)	15 (57.7)	14 (66.7)	0.5826	0.7288	0.3166	0.5292

1: Kruskal–Wallis was used for 3-group (Wilcoxon rank sum test if comparing pairs within a group, corrected α’ = α/[k(k-1)/2]). 2: Chi-square tests or Fisher’s test (corrected α’ = α/[k(k-1)/2] if comparing pairs within a group). Statistical significance was highlighted in italic. Wuhan: patients were infected in Wuhan; Wuhan-related: patients contracted the virus from Wuhan patients outside of Wuhan; non-Wuhan: patients acquired the disease outside of Wuhan without exposure to Wuhan patients.

### Clinical characteristics of COVID-19 patients with various viral exposure history

**Figure 2.**
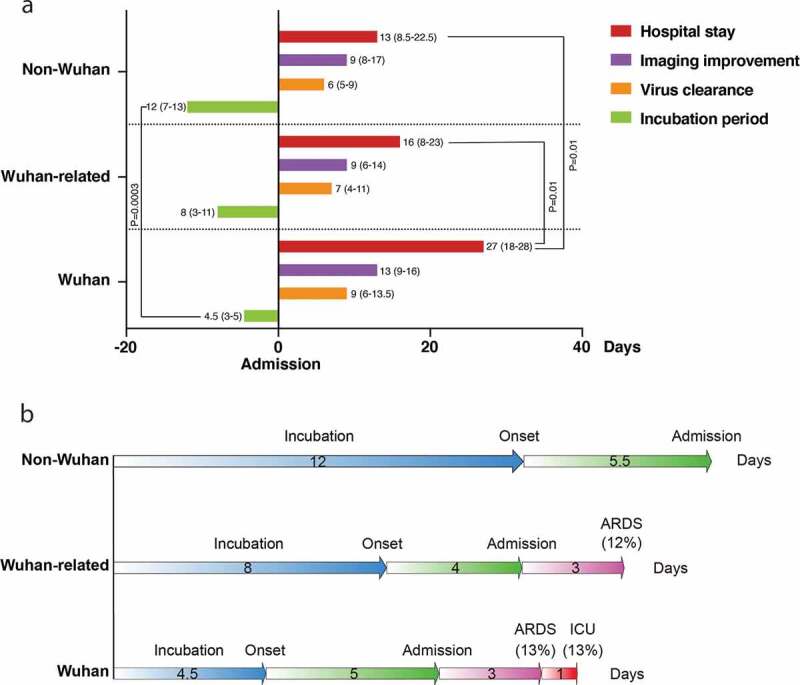
Clinical course of COVID-19 patients. (a) Duration of incubation period, virus clearance, image improvement, and hospital stay. (b) Timelines of clinical courses. Wuhan: patients were infected in Wuhan; Wuhan-related: patients contracted the virus from Wuhan patients; non-Wuhan: patients acquired the disease without exposure to Wuhan patients. ARDS: acute respiratory distress syndrome; ICU: intensive care unit.

There were more severe patients infected with the virus in Wuhan than those acquired the disease outside of Wuhan (Wuhan [53.3%], Wuhan-related [30.8%], non-Wuhan [14.3%], respectively) (Table 1, Figure 1(b)). The counts of total lymphocyte, T lymphocyte, CD4 and CD8 T lymphocyte in blood were lowest in Wuhan, lower in Wuhan-related and modest reduced in non-Wuhan patients. Comparing with non-Wuhan patients, CRP and IL-6 were dramatically elevated in Wuhan and increased in Wuhan-related groups. PaO_2_/FIO_2_ was lowest in Wuhan (206.03 ± 105.81), lower in Wuhan-related (297.25 ± 100.72), and normal in non-Wuhan (351.48 ± 38.12) patients (Table 2). Wuhan patients had highest respiratory failure (46.7%) comparing to Wuhan-related (19.2%) or non-Wuhan (0%) patients. Most patients received oxygen inhalation and anti-viral intervention with Lopinavir/Ritonavir or Abidol and aerosol inhalation of interferon α-2b. Treatment did not differ among these groups except more Wuhan patients received glucocorticoid and Gamma-globulin (Table 3). Duration of viral clearance appeared longer in Wuhan than either Wuhan-related or non-Wuhan group. It took Wuhan patients longer time to have chest CT image improved than Wuhan-related or non-Wuhan ones. Wuhan patients had much longer median hospital stay (27 days [18–28]) than either Wuhan-related (16 days [8–23]) or non-Wuhan patients (13 days [8.5–22.5]) ((Table 3, Figure 2). While the rest of the patients recovered and discharged by 17 March 2020, one died of respiratory failure and another died of probable glucocorticoid-associated gastro-intestinal bleeding in Wuhan group (13.3%) (Table 3). Multiple linear regression analysis estimated that every 1-day delay of imaging improvement increased 1.3-day hospital stay. Compared with Wuhan exposure, Wuhan-related exposure shortened 5.8-day hospital stay and non-Wuhan exposure for 8.6-day hospital stay (Table 4).

**Table 2.** Laboratory findings of COVID-19 patients with various viral exposure histories.

				P value			
	Wuhan [15]	Wuhan- related [26]	Non-Wuhan [21]	ANONA	Wuhan *vs* non-Wuhan	Wuhan *vs* Wuhan-related	Wuhan-related *vs* non-Wuhan
^1^White blood cell ×10^9^/L	6.28 (3.71–10.97)	5.46 (3.92–6.99)	4.64 (4.27–6.23)	0.4544	0.2824	0.3504	0.5279
^1^Neutrophil ×10^9^/L	5.7 (2.26–10.16)	3.4 (2.24–4.6)	2.84 (1.98–3.75)	0.1107	0.0522	0.1633	0.2944
^2^Total Lymphocyte ×10^9^/L	657.33 ± 374.27	865.7 ± 455.51	1468.3 ± 643.95	*0.0001*	*<0.05*	*<0.05*	*<0.05*
^2^T Lymphocyte/μL	461.69 ± 306.89	619.13 ± 383.67	1006.05 ± 378.68	*0.0002*	*<0.05*	*<0.05*	*<0.05*
^1^CD4 T Lymphocyte/μL	216 (129–397)	310 (118.5–470)	531 (368.5–715)	*0.0032*	*0.0021*	0.5963	*0.0065*
^2^CD8 T Lymphocyte/μL	170.64 ± 139.65	277.96 ± 158.85	444.55 ± 158.91	*0*	*<0.05*	*<0.05*	*<0.05*
^1^B Lymphocyte/μL	148 (78–166)	126 (65–144)	161 (106–246.5)	0.0814	0.0966	0.5435	0.0311
^1^NK Lymphocyte/μL	66 (32–179)	119.5 (45–256)	144 (114–232)	0.0981	0.0318	0.3464	0.1948
^1^C-reactive protein mg/L	26.9 (7–58.9)	10.4 (4.2–32)	4.9 (1.71–9.7)	*0.0197*	*0.0058*	0.1350	0.1309
^1^Interleukin-6 pg/mL	28.69 (5.91–44.02)	10.7 (5.72–21.6)	7.55 (5.45–13.74)	*0.0335*	*0.0130*	0.0529	0.3814
^1^Procalcitonin ng/mL	0.07 (0.04–0.1)	0.04 (0.03–0.09)	0.04 (0.04–0.06)	0.2033	0.0919	0.1364	0.5724
^2^PaO_2_/FIO_2_*	206.03 ± 105.81	297.25 ± 100.72	351.48 ± 38.12	*0.0008*	*<0.05*	*<0.05*	*<0.05*

1: Kruskal–Wallis was used for 3-group (Wilcoxon rank sum test if comparing pairs within a group, corrected α’ = α/[k(k-1)/2]). 2: ANOVA for three-group (SNK-q tests between-group comparisons). Statistical significance was highlighted in italic. Wuhan: patients were infected in Wuhan; Wuhan-related: patients contracted the virus from Wuhan patients; non-Wuhan: patients acquired the disease without exposure to Wuhan patients. *PaO_2_/FIO_2_: arterial partial pressure of oxygen/oxygen concentration. Data regarding the PaO_2_/FIO_2_ were missing for 20 patients (32.3%), of which 1 patient in Wuhan, 8 patients in Wuhan-related and 11 patients in non-Wuhan groups.

**Table 3.** Complications, treatments and outcome of COVID-19 patients with various viral exposure histories.

				P value			
	Wuhan [15]	Wuhan-related [26]	Non-Wuhan [21]	ANONA	Wuhan *vs* non-Wuhan	Wuhan *vs* Wuhan-related	Wuhan-related *vs* non-Wuhan
^1^Complication-no. (%)							
Septic shock	2 (13.3)	2 (7.7)	1 (4.8)	0.7152	0.5588	0.6149	1.0000
Respiratory failure	7 (46.7)	5 (19.2)	0 (0)	*0.0014*	*0.0008*	0.0834	0.0561
Acute respiratory distress syndrome	2 (13.3)	3 (11.5)	0 (0)	0.236	0.1667	1.000	0.2424
Acute cardiac injury	2 (13.3)	1 (3.8)	0 (0)	0.2464	0.1667	0.5427	1.0000
Acute kidney injury	0 (0)	2 (7.7)	0 (0)	0.505	1.0000	0.5244	0.4949
^1^Treatment-no. (%)							
Lopinavir/Ritonavir	14 (93.3)	20 (76.9)	10 (47.6)	*0.0095*	*0.0050*	0.2324	0.0659
Abidol	1 (6.7)	2 (7.7)	3 (14.3)	0.7502	0.6257	1.0000	0.6441
Interferon α-2b	12 (80.0)	23 (88.5)	13 (61.9)	0.0835	0.2951	0.6509	0.0433
Antibiotic	11 (73.3)	15 (57.7)	7 (33.3)	0.0502	0.0409	0.5020	0.1428
Glucocorticoid	7 (46.7)	5 (19.2)	2 (9.5)	*0.0393*	*0.0155*	0.0534	0.4364
Gamma-globulin	7 (46.7)	5 (19.2)	2 (9.5)	*0.0393*	*0.0155*	0.0534	0.4364
Oxygen inhalation	14 (93.3)	23 (88.5)	19 (90.5)	1.0000	1.0000	1.0000	1.0000
Noninvasive ventilation	2 (13.3)	3 (11.5)	1 (4.8)	0.6447	0.5588	1.0000	0.6174
Invasive mechanical ventilation	2 (13.3)	1 (3.8)	0 (0)	0.2464	0.1667	0.5427	1.0000
^1^Admission to intensive care unit-no. (%)	2 (13.3)	0 (0)	0 (0)	0.0555	0.1667	0.1280	NA
^2^Virus clearance, days	9 (6–13.5)	7 (4–11)	6 (5–9)	0.2218	0.3480	0.1132	0.7718
^2^Imaging improvement-no. (%)	13 (9–16)	9 (6–14)	9 (8–17)	0.3323	0.3257	0.1557	0.5793
^2^Hospital stay, days	27 (18–28)	16 (8–23)	13 (8.5–22.5)	*0.0187*	*0.0121*	*0.0121*	0.7662
^1^Discharge-no. (%)	13 (86.7)	26 (100)	21 (100.0)	0.0555	0.1667	0.128	NA
^1^Death-no. (%)	2 (13.3)	0 (0)	0 (0)	0.0555	0.1667	0.128	NA

1: Chi-square tests or Fisher’s test (corrected α’ = α/[k(k-1)/2] if comparing pairs within a group). 2: Kruskal–Wallis was used for three-group (Wilcoxon rank sum test if comparing pairs within a group, corrected α’ = α/[k(k-1)/2]). Statistical significance was highlighted in italic. Wuhan: patients were infected in Wuhan; Wuhan-related: patients contracted the virus from Wuhan patients; non-Wuhan: patients acquired the disease without exposure to Wuhan patients.

**Table 4.** Association of hospital stay with viral exposure history, viral clearance, and imaging improvement.

Variable	Parameter Estimate	Standard Error	P value
virus clearance	0.18430	0.22023	0.1476
imaging improvement	1.25448	0.15914	*<.0001*
Viral exposure			
Wuhan	Reference		
Wuhan-related	−5.79949	2.14614	*0.0093*
Non-Wuhan	−8.60479	2.22145	*0.0003*

Multiple linear regression analysis estimated the effects of viral exposure history, virus clearance, and imaging improvement on hospital stay. Statistical significance was highlighted in italic. Wuhan: patients were infected in Wuhan; Wuhan-related: patients contracted the virus from Wuhan patients; non-Wuhan: patients acquired the disease without exposure to Wuhan patients.

### Clinical differences between severe and nonsevere COVID-19 patients

**Figure 3.**
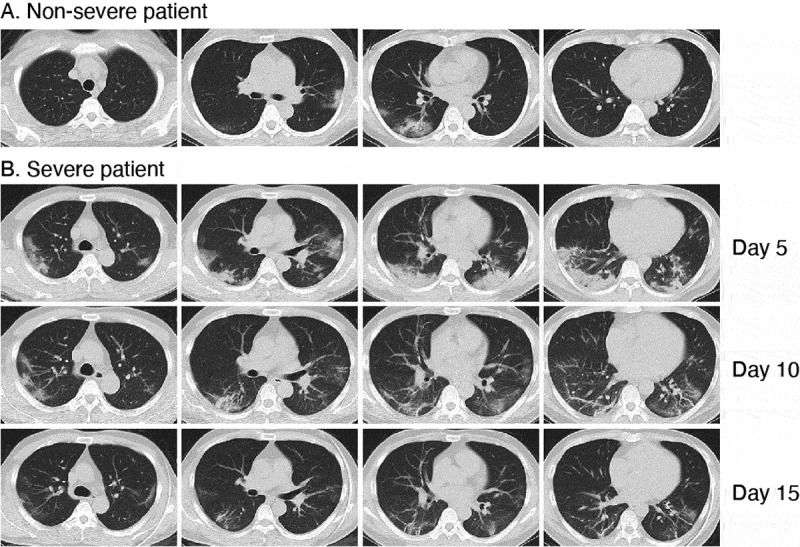
Chest computed tomographic images of COVID-19 patient. (a) Mild ground glass shadow in both lungs of nonsevere patient. (b) Obvious ground glass shadow in both lungs on day 5 of illness, ground glass shadow absorption on day 10 and lesion dissipation on day 15 after the treatment of a severe patient.

Of the 62 patients, 43 (69%) were diagnosed as mild and 19 (31%) as severe on admission. Comparing with nonsevere patients, severe patients were older, and more likely had hypertension and diabetes (Table 5). Severe patients had more WBCs and neutrophils, higher D-dimer, CRP, IL-6, PCT and lactic acid, and fewer lymphocytes (Table 6). The ratio of PaO_2_/FiO_2_ was 205.5 (117–262) mmHg in severe patients and 350.7 (324–384) mmHg in nonsevere patients. More severe patients had expectoration and dyspnea. Chest CT scan showed obviously bilateral ground glass opacity and patchy shadows in severe patients (Figure 3, Table 6). They developed septic shock, respiration failure, acute respiratory distress syndrome, acute cardiac injury, and had higher mortality rate. Antibiotics, glucocorticoid, gamma-globulin, noninvasive ventilation, and invasive mechanical ventilation were often used for severe patients (Table 6). After treatment, most of the patients recovered and their inflammation indicators were gradually back to normal ranges.

**Table 5.** Baseline characteristics and symptoms of COVID-19 patients.

	Total (62)	Severe [19]	Nonsevere (43)	*P* value
Age-yr (%)				
0–49	33 (53.2)	4 (21.1)	29 (67.4)	*0.0007*
≥50	29 (46.8)	15 (79.0)	14 (32.6)	
Sex-no. (%)				
Female	28 (45.2)	10 (52.6)	18 (41.9)	0.4321
Male	34 (54.8)	9 (47.4)	25 (58.1)	
Comorbidities-no. (%)				
Respiratory disease	4 (6.5)	2 (10.5)	2 (4.7)	0.5797
Hypertension	16 (25.8)	9 (47.4)	7 (16.3)	*0.0247*
Diabetes	9 (14.5)	6 (31.6)	3 (7.0)	*0.0189*
Cardiovascular disease	4 (6.5)	3 (15.8)	1 (2.3)	0.0816
Chronic kidney disease	2 (3.2)	2 (10.5)	0 (0)	0.0904
Symptoms-no. (%)				
Fever	55 (88.7)	16 (84.2)	39 (90.7)	0.6653
Fatigue	24 (38.7)	11 (57. 9)	13 (30.2)	*0.0392*
Cough	38 (61.3)	14 (73.7)	24 (55.8)	0.1829
Expectoration	16 (25.8)	10 (52.6)	6 (14.0)	*0.0033*
Pharyngalgia	13 (21.0)	3 (15.8)	10 (23.3)	0.7369

Chi-square tests or Fisher’s test (corrected α’ = α/[k(k-1)/2] if comparing pairs within a group). Statistical significance was highlighted in italic.

**Table 6.** Laboratory and radiographic findings of severe and nonsevere COVID-19 Patients.

		Median	
	Severe [19]	Nonsevere (43)	*P* Value
^1^White blood cell ×10^9^/L	8.2 (5.8–10.5)	4.4 (3.7–5.8)	*<0.0001*
^1^Neutrophil ×10^9^/L	7.3 (4.7–9.8)	2.4 (1.9–3.4)	*<0.0001*
^2^Lymphocytes ×10^9^/L	479.8 ± 365.8	1388.7 ± 576.0	*<0.0001*
^1^Monocyte ×10^9^/L	0.32 (0.2–0.5)	0.38 (0.2–0.5)	0.1324
^2^Platelet ×10^9^/L	196.1 ± 68.4	184.5 ± 55.8	0.4868
^1^Alanine aminotransferase U/L	34 (15–72)	22 (14–36)	0.1621
^1^Aspartate aminotransferase U/L	33 (23–70)	25 (21–31)	0.0687
^1^Creatinine μmol/L	78 (67–85)	79 (66–87)	0.8546
^1^Blood urea nitrogen mmol/L	5.5 (3.2–6.3)	3.8 (3.2–4.9)	*0.0413*
^1^Prothrombin time s	12.6 (11.4–13.8)	12.1 (11.5–12.6)	0.2857
^1^D-dimer mg/L	1.3 (0.5–5.0)	0.24 (0.2–0.4)	*<0.0001*
^1^Lactic acid mmol/L*	3.2 (2.3–3.5)	1.9 (1.5–2.2)	*0.0011*
^1^C-reactive protein mg/L	32 (9.3–69.1)	6.8 (2.3–10.7)	*0.0002*
^1^Interleukin-6 pg/mL	30.8 (14.6–55.1)	8.1 (5.7–16.4)	*0.0007*
^1^Procalcitonin ng/mL	0.09 (0.05–0.14)	0.04 (0.03–0.06)	*0.0003*
^1^PaO_2_/FIO_2_, mmHg¶	205.5 (117–262)	350.7 (324–384)	*<0.0001*
^3^Ground-glass opacity-no. (%)	18 (94.7)	29 (67.4)	*0.0247*
^3^Bilateral patchy shadowing-no. (%)	18 (94.7)	22 (51.2)	*0.0006*

1: Wilcoxon rank sum test. 2: *t* test. 3: Chi-square tests. Statistical significance was highlighted in italic. *Data regarding lactic acid were missing for seven patients (11.3%), which all occurred in nonsevere patients. ¶Data regarding the ratio of the partial pressure of arterial oxygen to the fraction of inspired oxygen (PaO_2_/FIO_2_) were missing for 20 patients (32.3%), which all occurred in non-severe patients.

### Discussion

This retrospective cohort study documented the epidemiologic and clinical features of 62 COVID-19 patients treated in a medical center in Beijing of China. Compared with COVID-19 patients contracted the virus directly or indirectly from Wuhan-exported patients outside Wuhan, patients infected with the virus in Wuhan have shorter incubation period, severe clinical presentation, and worse prognosis. Severe patients are also associated with older age and comorbidities.

COVID-19 is a respiratory viral disease presented with fever, dry cough, shortness of breath, and pneumonia. Although most patients have mild symptoms, the disease can become very serious and even deadly for elderly people with preexisting health conditions [2,12–15]. Globalization renders the world more vulnerable to the spread of infectious diseases. SARS-CoV-2 can be spread by asymptomatic, presymptomatic, and symptomatic carriers primarily via respiratory droplets during close face-to-face contact and therefore this virus spreads easily and sustainably in the community [16]. This ongoing pandemic was first documented in Wuhan City, Hubei Province, China. There are now much more confirmed cases and death outside of China than inside of China [6]. Many of published reports since the outbreak are about the patients contracted the virus inside Wuhan [2,12,17]. There are no comparison reports of the clinical manifestations and outcomes between early infected patients and later infected ones in same medical setting(s). We therefore investigated the potential distinct clinical features among the patients from Wuhan and outside Wuhan treated at our Hospital. Our data indicate that patients contracted the virus outside Wuhan had longer incubation period, milder disease, better response to treatment, shorter hospital stay, and superior clinical outcome. As of 30 April 2020, Chinese Center for Disease Control and Prevention reported that Wuhan City’s case death rate is 7.7% (3,869/50,333) compared with 2.3% (774/34,040) in the rest of the country [7]. Mizumoto and Chowell estimated that risk for death was up to 12% in Wuhan and 1% in the rest of China [14]. Even though flood of patients who overwhelmed the medical capacity certainly partially contributed to high death rate during the initial outbreak in Wuhan, all of the patients in this study were treated at the same hospital in Beijing. The duration of disease onset and hospital admission should reflect whether patients receive timely treatment. Since all of our patients were admitted 4–5.5 days by average after disease onset, Wuhan patients’ more serious condition was not attributed to delayed treatment. It is plausible that the patients exported from Wuhan might be less severe than those treated inside Wuhan. Nevertheless, two out of 15 (13.3%) Wuhan patients died while none of the 47 patients infected outside Wuhan died in this study. These data suggest that early generations of the viruses may be more potent than later descendants of the viruses.

COVID-19 shares many similarities with Middle East Respiratory Syndrome (MERS) and SARS [18,19]. Like MERS-CoV and SARS-CoV, sequencing analysis revealed mutations and deletions on coding and non-coding regions of SARS-CoV-2 viruses and suggested genetic diversity and rapid evolution of SARS-CoV-2 viruses [18,20]. Genetic variants might influence the clinical presentation and spread of the disease [21] . It is plausible that potent variants are lethal to the hosts and therefore are less transmissible and eventually eliminated. On the other hand, less potent mutants cause mild disease and are easily transmitted person-to-person in the community. Therefore, potential genetic drift to less severe variant may be responsible for mild clinical outcome for patients infected with the virus outside of Wuhan in China. Viral samples should be sequenced to elucidate potential genetic alterations which may be responsible for their reduced virulence. SARS-CoV-2 virus can be transmitted quite efficiently by affected people who are just mildly ill or even asymptomatic or presymptomatic [12,22–24]. Since MERS and SARS were spread only by symptomatic people with much less efficiency [25,26], COVID-19 is more contagious and much harder to contain [19]. Even though MERS and SARS have much higher case mortality rate, COVID-19 nevertheless has claimed much more lives overall. Corresponding counter measurements should be in place to combat this devastating pandemic.

Immune and inflammatory responses are prevalent in patients with initial viral and secondary bacteria and/or fungal infections. Lymphocytopenia is more prominent in Wuhan-exported patients and severe patients. Adaptive immune system may be either suppressed or not activated during viral infection. The virus may destroy lymphocytes and/or lymphatic organs. T lymphocyte damage may contribute to disease deterioration [27]. The patients who contracted the virus in Wuhan or over 50 with hypertension and diabetes were more likely to become severe patients.

With no specific antiviral treatment for COVID-19, our management for mild patients was mainly supportive care. For severe patients, we repurposed existing antiviral medications and used prophylactic antibiotics to prevent complication. 96.8% patients were recovered and discharged by 17 March 2020. Prospectively, remdesivir and dexamethasone may hold promise for COVID-19 treatment [28–33]. Vaccination of general population is vital for prevention of recurrent COVID-19 pandemic.

Taken advantage of the same medical setting, we provide evidence that primary infected COVID-19 patients are more severe than secondary transmitted patients. We suggest that lymphocytopenia is a biomarker for poor prognosis. Older patients with comorbidities should be treated prophylactically for potential complications. Because our data were drawn from hospitalized patients, they would not necessarily be generalized to the public at large. This study of the limited number of patients only offers a glimpse at clinical manifestations of Covid-19, our preliminary findings should therefore be interpreted with caution. To facilitate preventing and mitigating this unprecedented pandemic, our findings are warranted validation from multicenter epidemiologic and clinical studies. Many patients with asymptomatic or minor manifestation were likely not documented. Due to their long latency, these undocumented patients may contribute to the rapid and extensive dissemination of SARS-CoV2 [23,24]. Extensive screening for virus carriers, contact tracing, face mask wearing, hand hygiene, and social distancing should be imposed on general public to contain viral transmission in community before the arrival of long-lasting measures, such as vaccination [34]. Underlying mechanism responsible for putative reduction of virulence associated with ensuing infections should be dissected.
